# Supplementary material for: Transient loss of Polycomb components induces an epigenetic cancer fate
Source: Nature. 2024 Apr 24;629(8012):688–96. doi: 10.1038/s41586-024-07328-w (PMC11096130; doi:10.1038/s41586-024-07328-w)
Supplement: Supplementary file 1 — This file contains full descriptions for Supplementary Tables 1–6 and Supplementary Videos 1–2 (Tables and Videos supplied separately), and Supplementary Fig. 1, which contains the raw western blot data corresponding to Fig. 1b and Extended Data Fig. 1e. [file 41586_2024_7328_MOESM1_ESM.pdf]

nature portfolio

https://doi.org/10.1038/s41586-024-07328-w

## Supplementary information

# Transient loss of Polycomb components induces an epigenetic cancer fate

In the format provided by the authors and unedited

# Transient loss of Polycomb components induces an epigenetic cancer fate


## Authors

Parreno, V.[1*], Loubiere, V.[1,2*], Schuettengruber, B.[1], Fritsch, L.[1], Rawal, C. C.[3]. Erokhin, M.[4], Győrffy, B.[5,6], Normanno, D.[1], Di Stefano, M.[1], Moreaux, J.[7,8], Butova, N. L.[3], Chiolo, I.[3], Chetverina, D.[4], Martinez, A-M.[1#] & Cavalli, G.[1#]

## Affiliations

[1] Institute of Human Genetics, CNRS, University of Montpellier, Montpellier, France
[2] Research Institute of Molecular Pathology, Vienna BioCenter, Campus-Vienna-BioCenter 1, Vienna, Austria
[3] University of Southern California, 1050 Childs Way, RRI203, 90089, Los Angeles, CA
[4] Institute of Gene Biology, Russian Academy of Sciences, 34/5 Vavilov Street, Moscow, 119334 Russia
[5] Semmelweis University Department of Bioinformatics, 7-9 Tuzolto Street, H-1094, Budapest, Hungary
[6] Department of Biophysics, Medical School, University of Pécs, 12 Szigeti Street, H-7624, Pécs, Hungary
[7] Department of Biological Hematology, CHU Montpellier, 34295 Montpellier, France
[8] UFR Medicine, University of Montpellier, 34003 Montpellier, France

[*] These authors contributed equally to this work

# Corresponding authors: anne-marie.martinez@igh.cnrs.fr (A.M.M.) and giacomo.cavalli@igh.cnrs.fr (G.C.)

## Supplementary information

This manuscript includes supplementary information, namely Supplementary Figure 1, six Supplementary tables and two Supplementary Videos. *Note that excel tables could not be formatted in pdf and are provided as .xlsx files. Videos are provided as separate files as well.*

**FILE INDEX:**

**Supplementary Figure 1: Original images of Western Blots**
**a-** Raw image corresponding to the three biological replicates used for the Western blot analysis of PH protein levels in the eye precursor tissues of L3 larvae subjected to no ph-KD (control), constant or transient ph-KD at L1 stage (see Figure 1 b and Extended Data Fig. 1d). Lanes are labelled to guide the interpretation. Note that the figure has been horizontally flipped in order to show the most logical lane order. **b-** Raw images corresponding to the two biological replicates used for the Western blot analysis of PH protein levels after early L3 EDs were subjected to 24h of white-KD (w-KD, control) or ph-KD followed by 0h, 24h, 48h, 72h and 96h of recovery at 18°C (see Extended DataFig. 1e). This time course illustrates acute depletion and allows visualization of the kinetics of PH recovery after ph-KD. Lanes are labelled in the top part of the blot for clarity. **c-** Raw image corresponding to the three biological replicates used for the Western blot analysis of ZFH1 protein levels in the eye precursor tissues of L3 larvae subjected to no ph-KD (control), constant or transient ph-KD at L1 stage (see Extended Data Fig. 3e). Lanes are labelled to guide the interpretation. A short exposure is shown on the left and a longer exposure is shown in the right panel. Note that the images have been horizontally flipped in order to show the most logical lane order.

**Supplementary table 1: Differential analyses and FPKMs of no *ph*-KD, transient *ph*-KD and constant *ph*-KD (fly line with conditional GFP expression).**
For each *ph*-KD/*w*-KD comparison, DESeq2 outputs are shown on separated sheets. The “diff” column specifies whether the gene was considered as unaffected, upregulated or downregulated according to the following criteria: | log2FC | > 1 and padj<0.05. The last sheet contains, for each condition, the mean FPKMs across three biological replicates. Two-sided Wald test p-values were corrected for multiple testing using the FDR method (see DESeq2 documentation).

**Supplementary table 2: Clustering of differentially expressed genes, PcG binding and recovery status.**
For each gene, its corresponding cluster is shown (Figure 2b, “cluster” column). Genes for which ≥50% of the gene body overlaps a H3K27me3 repressive domain in control condition (no *ph*-KD) were considered as direct PcG targets (see the “PcG_bound” column). PcG-bound genes were further classified as “Irreversible”, “Reversible” or “Unaffected” based on their differential expression after constant and transient *ph*-KD (see the “class” column). NA means that the variable is irrelevant for the corresponding gene.

**Supplementary table 3: Clustering of differentially accessible ATAC-Seq peaks.**
For each ATAC-Seq peak detected after no *ph*-KD (control), constant or transient *ph*-KD, the corresponding cluster is reported in the “cluster” column (see Figure 4a). The ID of each peak corresponds to its dm6 genomic coordinates.

**Supplementary table 4: Differential analyses and FPKMs of *gfp*-KD, *zfh1*-KD and *Stat92E*-KD transcriptomes in combination with *w*-KD (control) or *ph*-KD.**
For each combination of RNAi, DESeq2 outputs are shown on separated sheets. The “diff” column specifies whether the gene was considered as unaffected, upregulated or downregulated according to the following criteria: | log2FC | > 1 and padj<0.05. The last sheet contains, for each condition, the mean FPKMs across three biological replicates. Two-sided Wald test p-values were corrected for multiple testing using the FDR method (see DESeq2 documentation).

**Supplementary table 5: Differential analyses and FPKMs of no *ph*-KD, transient *ph*-KD and constant *ph*-KD (fly line with constitutive GFP expression).**
For each *ph*-KD/*w*-KD comparison, DESeq2 outputs are shown on separated sheets. The “diff” column specifies whether the gene was considered as unaffected, upregulated or downregulated according to the following criteria: | log2FC | > 1 and padj<0.05. Two-sided Wald test p-values were corrected for multiple testing using the FDR method (see DESeq2 documentation).

**Supplementary table 6: Tissue area measurements and number of tissues analysed in immunofluorescence experiments.**

This table reports the areas (calculated in $\mu m^2$) for all tissues measured in the experiments reported in Figures 1e, Extended Data Fig. 1m, 5b and 5f, each one in a separate table sheet. A fifth sheet (called "All IF sample numbers) reports the number of samples (EDs or tumors) imaged for each of the immunostaining experiments. The corresponding figure numbers are indicated in column A and the number of samples used for analyses in each of the figures and conditions is indicated in the corresponding cells.

**Supplementary Video 1: EdU incorporation in a control early L3 eye disc.**

The Eye Disc showed in this video is a representative example of EdU incorporation in control Eye Discs. The tissue was prepared from a larva submitted to a 24h *white*-KD at 29°C followed by a 24h recovery at 18°C. EdU was incorporated and stained as described in the Methods. For microscopy, the disc was mounted in Vectashield and images were acquired using a 20X objective and a Zeiss Airyscan microscope, with a Z-step between sections of 0.30 μm. The video shows a stack of 66 sections that is rotated and zoomed in order to appreciate the different cell layers. EdU is primarily incorporated in the antennal part (left) and in the proliferating part of the eye disc. Little incorporation is observed in the posterior of the eye disc (right side).

**Supplementary Video 2: EdU incorporation upon a 24h *ph*-KD in an early L3 eye disc, followed by 24h recovery.**

The Eye Disc showed in this video is a representative example of EdU incorporation upon a transient *ph*-KD. The tissue was prepared from a larva submitted to a 24h *ph*-KD at 29°C followed by a 24h recovery at 18°C. EdU was incorporated and stained as described in the Methods. For microscopy, the disc was mounted in Vectashield and images were acquired using a 20X objective and a Zeiss Airyscan microscope, with a Z-step between sections of 0.30 μm. The Video shows a stack of 67 sections that is rotated and zoomed in order to appreciate the different cell layers. As opposed to localized incorporation observed in controls as illustrated in Figure 1f and Extended data Video 1, EdU is incorporated throughout the tissue.

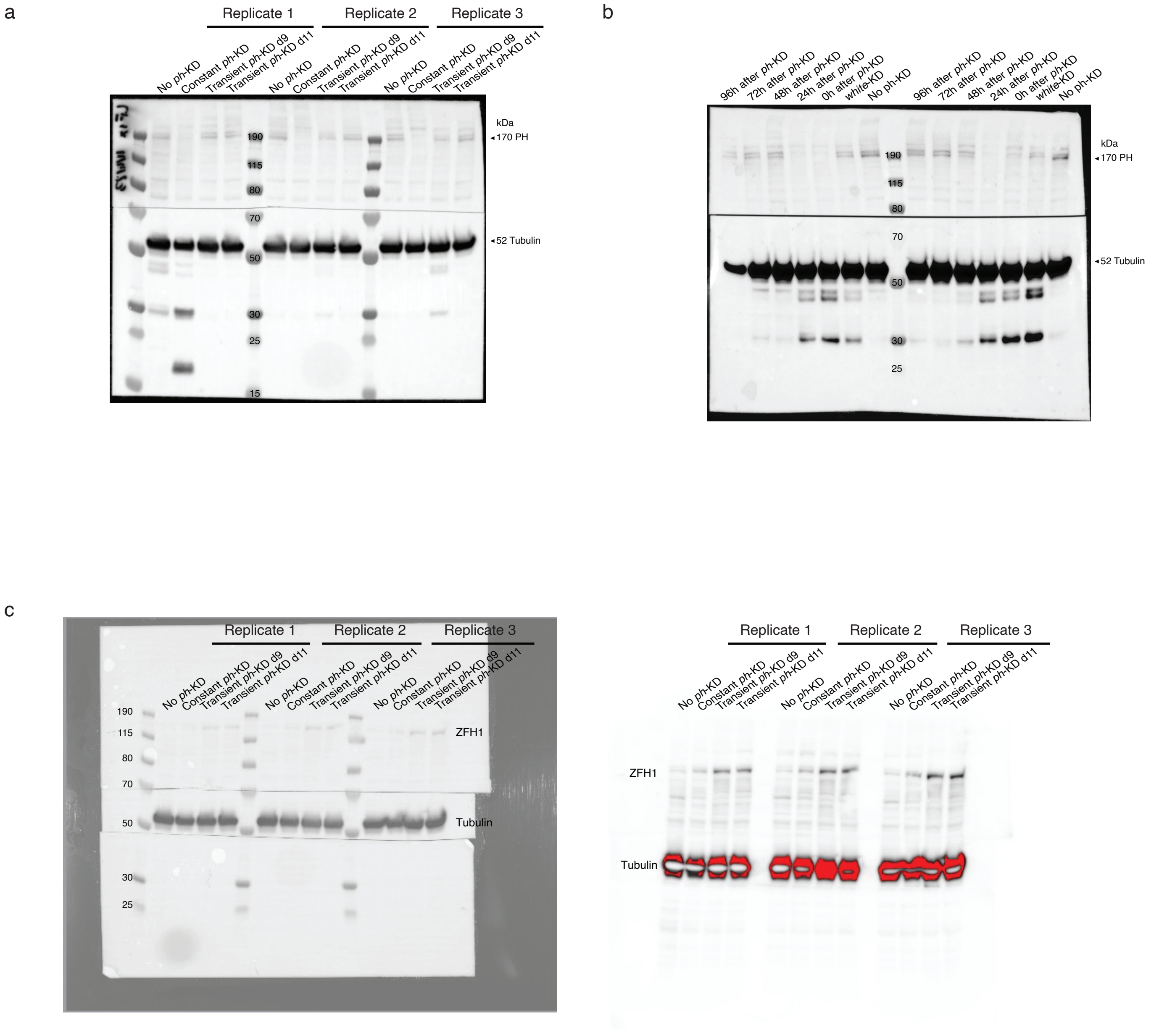


## Supplementary Figure 1: Original images of Western Blots

**a-** Raw image corresponding to the three biological replicates used for the Western blot analysis of PH protein levels in the eye precursor tissues of L3 larvae subjected to no ph-KD (control), constant or transient ph-KD at L1 stage (see Figure 1 b and Extended Data Fig. 1d). Lanes are labelled to guide the interpretation. Note that the figure has been horizontally flipped in order to show the most logical lane order. **b-** Raw images corresponding to the two biological replicates used for the Western blot analysis of PH protein levels after early L3 EDs were subjected to 24h of white-KD (w-KD, control) or ph-KD followed by 0h, 24h, 48h, 72h and 96h of recovery at 18°C (see Extended Data Fig. 1e). This time course illustrates acute depletion and allows visualization of the kinetics of PH recovery after ph-KD. Lanes are labelled in the top part of the blot for clarity. **c-** Raw image corresponding to the three biological replicates used for the Western blot analysis of ZFH1 protein levels in the eye precursor tissues of L3 larvae subjected to no ph-KD (control), constant or transient ph-KD at L1 stage (see Extended Data Fig. 3e). Lanes are labelled to guide the interpretation. A short exposure is shown on the left and a longer exposure is shown in the right panel. Note that the images have been horizontally flipped in order to show the most logical lane order.
