# Supplementary material for: Transient loss of Polycomb components induces an epigenetic cancer fate
Source: Nature. 2024 Apr 24;629(8012):688–96. doi: 10.1038/s41586-024-07328-w (PMC11096130; doi:10.1038/s41586-024-07328-w)
Supplement: Supplementary file 2 — Reporting Summary [file 41586_2024_7328_MOESM2_ESM.pdf]

nature portfolio

Corresponding author(s): Anne-Marie Martinez and Giacomo Cavalli

Last updated by author(s): Jan 9, 2024

Reporting Summary

Nature Portfolio wishes to improve the reproducibility of the work that we publish. This form provides structure for consistency and transparency in reporting. For further information on Nature Portfolio policies, see our Editorial Policies and the Editorial Policy Checklist.

Statistics

For all statistical analyses, confirm that the following items are present in the figure legend, table legend, main text, or Methods section.

n/a	Confirmed	
☐	☒	The exact sample size (*n*) for each experimental group/condition, given as a discrete number and unit of measurement
☐	☒	A statement on whether measurements were taken from distinct samples or whether the same sample was measured repeatedly
☐	☒	The statistical test(s) used AND whether they are one- or two-sided<br>*Only common tests should be described solely by name; describe more complex techniques in the Methods section.*
☒	☐	A description of all covariates tested
☐	☒	A description of any assumptions or corrections, such as tests of normality and adjustment for multiple comparisons
☐	☒	A full description of the statistical parameters including central tendency (e.g. means) or other basic estimates (e.g. regression coefficient) AND variation (e.g. standard deviation) or associated estimates of uncertainty (e.g. confidence intervals)
☐	☒	For null hypothesis testing, the test statistic (e.g. *F*, *t*, *r*) with confidence intervals, effect sizes, degrees of freedom and *P* value noted<br>*Give P values as exact values whenever suitable.*
☒	☐	For Bayesian analysis, information on the choice of priors and Markov chain Monte Carlo settings
☐	☒	For hierarchical and complex designs, identification of the appropriate level for tests and full reporting of outcomes
☐	☒	Estimates of effect sizes (e.g. Cohen's *d*, Pearson's *r*), indicating how they were calculated

Our web collection on statistics for biologists contains articles on many of the points above.

Software and code

Policy information about availability of computer code

Data collection	All immunostaining images were acquired using a Leica SP8 confocal microscope. Images of EdU stained-eye discs were acquired using a Zeiss LSM980 Airyscan microscope in 4Y modality. RT-qPCR were performed using LightCycler480. Western Blot images were acquired using the ChemiDoc Imaging Systems, Bio-Rad. To score tumor progression in allografts, flies were imaged every two days using Leica MZ FLIII in order to verify GFP as a readout of tumor growth. Injected Drosophila pictures were taken using Ximea USB 3.1 Gen1 camera with a Sony CMOS-xiCAll sensor. NGS illumina sequencing was performed on a Novaseq 6000.
Data analysis	Immunostaining were analysed using Fiji version 1.54h. Airyscan images of EdU stained-eye discs were processed with ZEN (version 3.6 Blue Edition, Zeiss) using default settings. Western Blot gels were analysed using ImageLab software version 6.1 from Bio-Rad. EdU movies were created using Imaris (version 10.1, Oxford Instruments). RT-qPCR were analysed using LightCycler software version 1.5.1. Alignments were performed using Burrows-Wheeler Aligner (BWA, gDNA), bowtie2 (CUT&RUN, ChIP-Seq, ATAC-Seq, v2.3.5.1) and the Rsubread R package (v2.0.1). Peak calling was performed using MACS2 (v2.2.7.1). Differential analysis of gene expression was performed using the DESeq2 R package (v1.26.0). Motif analysis was performed using the i-cisTarget online tool (database v.6.0). Genomic DNA variants were called using the GATK software. For Multiple Myeloma meta analysis, clustering was performed using the Morpheus software and violin plots using GraphPad Prism. Difference in overall survival between groups of patients was assayed with a log-rank test and survival curves plotted using the Kaplan-Meier method (Maxstat R Package). All other in-house bioinformatic analyses of Drosophila data were performed in R version 4.2.0, and full custom code were made publicly available at https://github.com/vloubiere/Parreno_Loubiere_2023.

For manuscripts utilizing custom algorithms or software that are central to the research but not yet described in published literature, software must be made available to editors and reviewers. We strongly encourage code deposition in a community repository (e.g. GitHub). See the Nature Portfolio guidelines for submitting code & software for further information.

Data

Policy information about availability of data

All manuscripts must include a data availability statement. This statement should provide the following information, where applicable:

- Accession codes, unique identifiers, or web links for publicly available datasets
- A description of any restrictions on data availability
- For clinical datasets or third party data, please ensure that the statement adheres to our policy

All genomic data produced for this manuscript have been deposited on GEO repository under the public accession number GSE222193. All other data were retrieved from publicly available data as indicated.

Human research participants

Policy information about studies involving human research participants and Sex and Gender in Research.

Reporting on sex and gender	Not Applicable
Population characteristics	Not Applicable
Recruitment	Not Applicable
Ethics oversight	Not Applicable

Note that full information on the approval of the study protocol must also be provided in the manuscript.

Field-specific reporting

Please select the one below that is the best fit for your research. If you are not sure, read the appropriate sections before making your selection.

☒ Life sciences ☐ Behavioural & social sciences ☐ Ecological, evolutionary & environmental sciences

For a reference copy of the document with all sections, see nature.com/documents/nr-reporting-summary-flat.pdf

Life sciences study design

All studies must disclose on these points even when the disclosure is negative.

Sample size	Sample size was defined in compliance with the gold standards of the field, such that relevant statistical parameters (mean, median...) would get stabilized. ChIP-Seq, CUT&RUN and ATAC-Seq were performed in duplicates, following encode's standards (https://www.encodeproject.org/chip-seq/transcription_factor/#standards; https://www.encodeproject.org/atac-seq/#standards). RNA-Seq were performed in triplicates, following encode's recommandations (https://www.encodeproject.org/data-standards/rna-seq/long-rnas/).<br>For sample sizes of Immunostaining experiments, see Supplementary Table 6, sheet name "All IF sample numbers".<br>For the patients cohorts discussed in the extended data Fig 12, the publicly available TT2 cohort (n=345) enables detection of transcriptional patterns associated with therapy resistance and overall survival in Multiple Myeloma. Furthermore, five other independent cohorts of patients with Multiple Myeloma (n=158, n=282, n=188, n=63 and n=51) were used as validation datasets. The original papers describing each cohort, including discussions on the sample sizes are provided in the legends relative to extended data.
Data exclusions	For transcriptome differential expression analysis, only the genes with at least 10 counts across all tested conditions were retained, complying with good practices. Similarly, only the genes that were differentially expressed in any of the tested condition were retained for the clustering of differentially expressed genes. For ATAC-Seq data, only the peaks overlapping at least 100 reads across all tested condition were considered, to avoid weak or noisy peaks. Only the peaks showing significant changes in accessibility during tested condition and had a log10(baseMean) value bigger or equal to 1.25 were considered for clustering.
Replication	All experiments were performed several times on different days, and only consistent observations were reported (see replicates section for further details regarding biological replicates).<br>For transcriptomic, RT-qPCR and western blot analysis, experiments were performed in biological triplicates. ATAC-seq, Cut&RUN, ChIP-seq and immunostaining experiments were performed in biological duplicates. Each biological replicate was obtained from independent genetic crosses. The only exception was the phospho H2AV staining shown in Figure 1j and Extended Fig.2c, which was performed once, but scoring tissues coming from six independent genetic crosses. This is now indicated in the methods section of the manuscript.
Randomization	This study does not require proper randomization protocols. However, collected flies were always randomly selected to perform the different experiments.
Blinding	Blinding is not applicable here, since data anonymization is not compatible with quality controls and the identity of control samples must be known in order to perform genomic data analysis. However, reported data are based on unbiased analysis avoiding confirmation bias and/or

subjective assumption of qualitative data.

Reporting for specific materials, systems and methods

We require information from authors about some types of materials, experimental systems and methods used in many studies. Here, indicate whether each material, system or method listed is relevant to your study. If you are not sure if a list item applies to your research, read the appropriate section before selecting a response.

Materials & experimental systems

n/a	Involved in the study
☐	☒ Antibodies
☒	☐ Eukaryotic cell lines
☒	☐ Palaeontology and archaeology
☐	☒ Animals and other organisms
☒	☐ Clinical data
☒	☐ Dual use research of concern

Methods

n/a	Involved in the study
☐	☒ ChIP-seq
☒	☐ Flow cytometry
☒	☐ MRI-based neuroimaging

Antibodies

Antibodies used

Antibodies are described in the Methods section of the manuscript.

The following primary antibodies were used (Antibody, dilution, Provider, Catalogue number) for immunostaining:
- PH, 1:500,
- ELAV, 1:1000, DSHB, 9F8A9,
- ABD-B, 1:1000, DSHB, 1A2E9,
- GFP, 1:500, Invitrogen, A10262
- ZFH1, 1:2000
- Histone H2AvD pS137, 1:500, Rockland, 600-401-914
- Rhodamine phalloidin Alexa Fluor 555, 1:1000, Invitrogen, R415
- Rhodamine phalloidin Alexa Fluor 488, 1:1000, Invitrogen, A12379

The following secondary antibodies were used (Antibody, dilution, Provider, Catalogue number) for immunostaining:
- donkey anti-goat Alexa Fluor 555, 1:1000, Invitrogen, A-21432
- donkey anti-mouse Alexa Fluor 647, 1:1000, Invitrogen, A-31571
- donkey anti-chicken Alexa Fluor 488, 1:1000, Clinisciences, 703-546-155
- donkey anti-rabbit Alexa Fluor 555, 1:1000, Invitrogen, A-31572

The folowing antibodies were used (Antibody, dilution, Provider, Catalogue number) for western blot:
- PH, 1:200, gift from Renato Paro
- ZFH1, 1:2000, gift from Erika Bach
- Beta-tubulin, 1:5000, DSHB, AA12.1

The following HRP-conjugated secondary antibodies were used (Antibody, dilution, Provider, Catalogue number) for western blot:
- goat anti-rabbit, 1:15000, Sigma, A0545
- rabbit anti-mouse, 1:15000, Sigma, A9044

The following antibodies were used (Antibody, dilution, Provider, Catalogue number) for ChIP-seq:
- PH, 1:100, Giacomo Cavalli laboratory

The following antibodies were used (Antibody, dilution, Provider, Catalogue number) for CUT&RUN:
- H3K27me3, 1:100, Active motif, 39155
- H2AK118Ub, 1:100, Cell Signaling Technology, 8240S
- H3K27Ac, 1:100, Active motif, 39133
- IgG, 1:100, Cell Signaling Technology, 2729S

Validation

All commercial antibodies are validated for the use of immunofluorescence, western blot, ChIP-seq and CUT&RUN. Data are available on the manufacturer's website. The following antibodies have been validated by the manufacturer, by previous papers and/or in this paper by RNAi knockdown (Antibody, Validation and references):
- PH, immunostaining, PMID: 16530043, ChIP-seq PMID: 27643538, western blot validated in this paper by RNAi knockdown
- ELAV, immunotaining, Elav-9F8A9 was deposited to the DSHB by Rubin, Gerald M. (DSHB Hybridoma Product Elav-9F8A9), PMID: 8033205
- ABD-B, immunostaining, anti-ABD-B (1A2E9) was deposited to the DSHB by Celniker, S. (DSHB Hybridoma Product anti-ABD-B (1A2E9)), PMID: 2575066
- GFP, immunostaining, specificity of chicken-anti-GFP (#A10262, Invitrogen) has been verified in this paper by verifying that it produces signal at the expected molecular weight and only in fly lines and experimental conditions that lead to the presence of GFP.
- ZFH1, immunostaining PMID: 20412771
- Histone H2AvD pS137, validated in PMID: PMID: 29925946 for immunostaining, see also manufacturer's website for references
- Rhodamine phalloidin, immunostaining see manufacturer's website for references

- Beta-tubulin, AA12.1 was deposited to the DSHB by Walsh, C. (DSHB Hybridoma Product AA12.1), PMID: 6363422
- H3K27me3, CUT&RUN see manufacturer's website for references. Additional validation is provided in this manuscript, by verifying that CUT&RUN signal is lost upon knock down of the Polycomb component PH.
- H3K27Ac, CUT&RUN see manufacturer's website for references. Additional validation is provided in this manuscript, by verifying that CUT&RUN signal is gained at many Polycomb target genes that are induced upon knock down of the Polycomb component PH.
- H2AK118ub, CUT&RUN see manufacturer's website for references. Additional validation is provided in this manuscript, by verifying that CUT&RUN signal is lost upon knock down of the Polycomb component PH.

Animals and other research organisms

Policy information about studies involving animals; ARRIVE guidelines recommended for reporting animal research, and Sex and Gender in Research

Laboratory animals

For crosses adults were between 2-3 days old when crosses were performed. Larvae were between 9 to 11 days.
The following flies strain were used in this study :
eyFLP, Act5C (FRT.CD2) Gal4 ; + ; UAS-GFP (BL#64095), Tub-Gal80TS ; TM2/TM6B,Tb (BDSC#7019), UAS-ph RNAi (VDRC#50028), UAS-Psc RNAi (BL#38261), UAS-Su(z)2 RNAi (VDRC#100096), Sna[Sco] / CyO ; Tub-Gal80TS (BL#7018), UAS-white RNAi (BL#33623), UAS-gfp RNAi (BL#9331), UAS-zfh1 RNAi (VDRC#103205), UAS-Stat92E RNAi (VDRC#43866), ey-FLP (BL#5580), Ubi-p63E(FRT.STOP)Stinger (BL#32249), Act5C(FRT.CD2) Gal4 , UAS-RFP/TM3,Sb (BL#30558), His2Av-mRFP (BL#23650)

Wild animals

The study did not involve wild animals.

Reporting on sex

In order to standardize expression of the RNAi constructs, for all crosses, we used virgin females for all lines expressing GAL4.
All Drosophila analyzed in the study were females in order to avoid sex biases. Of note, male flies always showed similar phenotypes.

Field-collected samples

The study did not involve samples collected from the field.

Ethics oversight

This study was performed under the ethical approval N. n6906C2 of the MINISTÈRE DE L' ENSEIGNEMENT SUPÉRIEUR,
DE LA RECHERCHE ET DE L' INNOVATION, issued on April 8, 2020. Drosophila strains used and the age of the populations are reported in the manuscript and the methods section.

Note that full information on the approval of the study protocol must also be provided in the manuscript.

ChIP-seq

Data deposition

☒ Confirm that both raw and final processed data have been deposited in a public database such as GEO.

☒ Confirm that you have deposited or provided access to graph files (e.g. BED files) for the called peaks.

Data access links
May remain private before publication.

GSE222193

Files in database submission

For each ChIP-Seq data, bw genomic tracks and peak files are available. For each ATAC-Seq data, bw genomic tracks and peak files are available. For RNA-Seq, bw tracks, normalized counts and differential expression files are available. For gDNA sequencing, all raw SNPs and InDels as well as somatic variants are available. Finally, raw fastq files are available for all experiments.

Genome browser session
(e.g. UCSC)

A UCSC browser session is available at http://genome-euro.ucsc.edu/s/cavalli/EpiCancer and is mentioned in the Data availability Heading in the manuscript. Furthermore, at: https://www.ncbi.nlm.nih.gov/geo/query/acc.cgi?&acc=GSE222193 users can very easily download all bigwig (BW) format files that can the then simply dispayed in local genome browsers such as IGV or IGB that can be downloaded for free and used on any desktop computer.

Methodology

Replicates

For ChIP-Seq and Cut&Run data, two replicates were used per condition.

Sequencing depth

Condition Total reads Uniquely aligned reads
H2AK118Ub_PH18_rep1 5,461,997 3,491,769
H2AK118Ub_PH18_rep2 10,670,328 6,534,683
H2AK118Ub_PH29_rep1 16,931,039 5,085,514
H2AK118Ub_PH29_rep2 7,688,530 5,173,802
H2AK118Ub_PHD11_rep1 8,223,640 6,108,599
H2AK118Ub_PHD11_rep2 8,526,156 6,702,363
H2AK118Ub_PHD9_rep1 8,226,806 5,787,468
H2AK118Ub_PHD9_rep2 6,836,989 4,212,828
H3K27Ac_PH18_rep1 9,502,481 6,423,401
H3K27Ac_PH18_rep2 9,455,194 6,557,137
H3K27Ac_PH29_rep1 7,933,451 6,020,508
H3K27Ac_PH29_rep2 10,928,323 6,094,928

H3K27Ac_PHD11_rep1 10,837,602 8,099,891
H3K27Ac_PHD11_rep2 14,222,258 10,293,439
H3K27Ac_PHD9_rep1 11,714,006 7,742,745
H3K27Ac_PHD9_rep2 11,392,314 8,577,451
H3K27me3_PH18_rep1 9,860,407 7,139,641
H3K27me3_PH18_rep2 8,980,660 7,500,962
H3K27me3_PH29_rep1 7,800,260 5,501,836
H3K27me3_PH29_rep2 9,340,779 6,440,368
H3K27me3_PHD11_rep1 9,866,072 6,994,121
H3K27me3_PHD11_rep2 10,130,345 7,451,714
H3K27me3_PHD9_rep1 10,431,545 7,553,174
H3K27me3_PHD9_rep2 9,577,300 5,745,957
IgG_PH18_rep1 9,966,366 5,920,782
IgG_PH18_rep2 10,683,819 4,882,917
IgG_PH29_rep1 10,581,617 4,375,438
IgG_PH29_rep2 12,450,166 6,844,033
IgG_PHD11_rep1 9,081,016 5,772,603
IgG_PHD11_rep2 12,626,730 8,014,611
IgG_PHD9_rep1 8,807,714 4,619,458
IgG_PHD9_rep2 10,756,886 6,785,571
PH_PH18_rep1 11,937,832 7,640,526
PH_PH18_rep2 13,949,018 8,016,532
PH_PH29_rep2 11,382,596 6,293,104
PH_PHD11_rep1 13,554,060 9,296,764
PH_PHD11_rep2 17,309,927 10,419,882
PH_PHD9_rep1 14,598,749 8,370,146
PH_PHD9_rep2 14,144,138 8,201,413
input_PH18_rep1 10,263,837 7,869,566
input_PH18_rep2 14,283,477 10,695,459
input_PH29_rep1 10,939,021 6,402,253
input_PH29_rep2 12,758,267 10,242,311
input_PHD11_rep1 11,380,515 9,000,824
input_PHD11_rep2 15,150,197 12,146,663
input_PHD9_rep1 11,116,399 8,489,153

Antibodies

The following antibodies were used (Antibody, dilution, Provider, Catalogue number) for ChIP-seq :
- PH, 1:100, Giacomo Cavalli laboratory

The following antibodies were used (Antibody, dilution, Provider, Catalogue number) for CUT&RUN :
- H3K27me3, 1:100, Active motif, 39155
- H2AK118Ub, 1:100, Cell Signaling Technology, 8240S
- H3K27Ac, 1:100, Active motif, 39133
- IgG, 1:100, Cell Signaling Technology, 2729S

Peak calling parameters

Peak calling was performed using MACS2 using with the following parameters: --keep-dup 1 -g dm -f BAMPE -B --SPMR

Data quality

The quality of the data was assessed using PCA on normalized counts and Pearson's correlation coefficients between replicates. Following are the number of significant peaks found for each condition: H2AK118Ub_PH18 = 728, H2AK118Ub_PH29= 45, H2AK118Ub_PHD11= 217, H2AK118Ub_PHD9= 228, H3K27Ac_PH18= 3836, H3K27Ac_PH29= 4380, H3K27Ac_PHD11= 4871, H3K27Ac_PHD9= 4360, H3K27me3_PH18 = 265, H3K27me3_PH29= 161, H3K27me3_PHD11= 205, H3K27me3_PHD9= 251, PH_PH18= 2855, PH_PH29= 71, PH_PHD11= 2873, PH_PHD9= 2215

Software

Peaks calling was performed using MACS2, and further quantification were performed in R (v4.2.0). All custom code will be made publicly available on github before publication (yet to come).
